# Effect of galvanic vestibular stimulation applied at the onset of stance on muscular activity and gait cycle duration in healthy individuals

**DOI:** 10.3389/fncir.2022.1065647

**Published:** 2023-02-09

**Authors:** Faezeh Abbariki, Youstina Mikhail, Adjia Hamadjida, Jonathan Charron, Jean-Marc Mac-Thiong, Dorothy Barthélemy

**Affiliations:** ^1^School of Rehabilitation, Université de Montréal, Montreal, QC, Canada; ^2^Centre for Interdisciplinary Research in Rehabilitation of Greater Montreal (CRIR) and Centre Intégré Universitaire en Santé et Services Sociaux (CIUSSS) du Centre-Sud-de-l’Île-de-Montréal, Montreal, QC, Canada; ^3^Faculty of Medicine, Université de Montréal, Montreal, QC, Canada; ^4^Department of Live Science, Higher Teacher Training College of Bertoua, University of Bertoua, Bertoua, Cameroon; ^5^Centre Intégré Universitaire en Santé et Services Sociaux du nord de l’île de Montréal (CIUSSS NIM), Hôpital du Sacré-coeur de Montréal (HSCM), Montréal, QC, Canada

**Keywords:** human, locomotion, gait asymmetry, soleus, tibialis anterior, vestibular responses

## Abstract

Locomotion requires the complex involvement of the spinal and supraspinal systems. So far, the role of vestibular input in gait has been assessed mainly with respect to gait stability. The noninvasive technique of galvanic vestibular stimulation (GVS) has been reported to decrease gait variability and increase gait speed, but the extent of its effect on spatiotemporal gait parameters is not fully known.

**Objective:** Characterize vestibular responses during gait and determine the influence of GVS on cycle duration in healthy young participants.

**Methods:** Fifteen right-handed individuals participated in the study. Electromyography (EMG) recordings of the bilateral soleus (SOL) and tibialis anterior muscles (TA) were performed. First, to determine stimulation intensity, an accelerometer placed on the vertex recorded the amplitude of the head tilts evoked by the GVS (1–4 mA, 200 ms) to establish a motor threshold (T). Second, while participants walked on a treadmill, GVS was applied at the onset of the stance phase during the treadmill gait with an intensity of 1 and 1.5 T with the cathode behind the right (RCathode) or left ear (LCathode). EMG traces were rectified, averaged (*n* = 30 stimuli), and analyzed. Latency, duration, and amplitude of vestibular responses as well as the mean duration of the gait cycles were measured.

**Results:** GVS mainly induced long-latency responses in the right SOL, right TA and left TA. Only short-latency responses were triggered in the left SOL. Responses in the right SOL, left SOL and left TA were polarity dependent, being facilitatory with RCathode and inhibitory with LCathode, whereas responses in the right TA remained facilitatory regardless of the polarity. With the RCathode configuration, the stimulated cycle was prolonged compared with the control cycle at both 1 and 1.5 T, due to prolonged left SOL and TA EMG bursts, but no change was observed in right SOL and TA. With LCathode, GVS did not modify the cycle duration.

**Conclusion:** During gait, a brief, low-intensity GVS pulse delivered at the right stance onset induced mainly long-latency polarity-dependent responses. Furthermore, a RCathode configuration increased the duration of the stimulated gait cycle by prolonging EMG activity on the anodic side. A similar approach could be explored to influence gait symmetry in individuals with neurological impairment.

## Introduction

Locomotion requires dynamic interactions between spinal and supraspinal networks (Grillner and Dubuc, 1988; Rossignol et al., 2006). Many studies have described the involvement of supraspinal tracts in gait. Notably the corticospinal tract is involved in skilled locomotion and foot placement (Barthélemy et al., 2011) and vestibulo/reticulospinal tracts enable anticipatory and feedback balance control during gait (see Mackinnon, 2018). In humans, a growing amount of studies are reporting on the role of the vestibular system during gait use galvanic vestibular stimulation (GVS; Fitzpatrick et al., 1999).

GVS is a noninvasive method to stimulate electrically the peripheral vestibular system, namely the vestibular hair cells and irregular vestibular afferents (Goldberg et al., 1982, 1984; Norris et al., 1998). GVS induces responses in muscles that are active in a balance task (Fitzpatrick et al., 1994), mainly through vestibulospinal and reticulospinal tracts (Wilson et al., 1979; Peterson et al., 1981; Kennedy et al., 2004). In most studies, GVS is applied in a binaural, bipolar configuration when the cathode is on one side of the head and the anode on the other side (Fitzpatrick and Day, 2004; Lajoie et al., 2021). Such a configuration can increase the firing pattern of the irregular vestibular afferents on the cathode side and decrease it on the anode side (Fitzpatrick and Day, 2004). Responses are typically divided into short-latency response (SLR), medium-latency response (MLR), and long-latency response (LLR). The MLR is the most prominent and reproducible response and corresponds to the behavioral response, which is a tilt (lean) toward the anode side (Britton et al., 1993; Fitzpatrick et al., 1994).

Studies have used GVS to assess the role of vestibular input in gait mainly with respect to gait stability (Fitzpatrick and Day, 2004; St. George and Fitzpatrick, 2010). Input from the vestibular system is crucial for foot placement and determining the gait path trajectory (Bent et al., 2002, 2004). Furthermore, when GVS is applied during gait, it induces a deviation in the ongoing path, and participants stray toward the anodal side which is greatest for slower speeds of locomotion in controls (Fitzpatrick et al., 1999; Jahn et al., 2000; Bent et al., 2004). These effects were more important at the onset of stance, which suggests that the vestibular system plays a significant role in the stability of the limb during the swing-to-stance transition. However, the role of the vestibular system during gait is not fully known and the mechanisms by which GVS modulates the gait pattern would need further investigation.

First, GVS is likely to affect the spatiotemporal parameters of gait, but the nature of its effects remains unclear. Low-intensity noisy GVS, which consists of a noisy, alternating electrical current was shown to decrease variability in the gait cycle timing and trunk acceleration during perturbed treadmill walking (Wuehr et al., 2016a, b; Lajoie et al., 2021; McLaren et al., 2022). During overground walking with eyes closed, reduced variability in stride time and stride length have also been reported (Wuehr et al., 2016b; Iwasaki et al., 2018; Piccolo et al., 2020). Thus, variability in spatio-temporal parameters seemed to be reduced but the changes in the parameters themselves still need to be clarified.

Second, the effects of GVS were reported for the gait cycle as a whole, which might overlook side-dependent variations in the responses and might lead to an incomplete portrait of GVS effects on gait. Indeed, as the responses to bipolar binaural GVS depend on the side of the cathode/anode (Fitzpatrick and Day, 2004), the effects on the gait cycle might also differ between the two sides of the body.

Third, most studies apply nGVS continuously throughout the gait cycle to assess the modulation of spatio-temporal parameters (see Lajoie et al., 2021; McLaren et al., 2022). However, vestibular inputs might be required at specific phase of the gait cycle, mainly at heel strike or early stance (Bent et al., 2004; Iles et al., 2007). A more targeted stimulation at that phase of the gait cycle might be enough to lead to significant changes in the gait cycle.

Hence, in this study, we aimed to better understand the effect of a short-duration, bipolar and binaural GVS pulse applied at the onset of stance (10 ms after right heel contact) on the EMG activity during ongoing walking and on the gait cycle duration. We hypothesized that such stimulation would modify the gait pattern and the duration of the gait cycle differentially between the right and left lower limbs. More specifically, when applying a binaural, bipolar GVS, the gait cycle could be either prolonged or shortened on one side without affecting the other side. If indeed that is confirmed, our findings could be clinically relevant and be at the base of a therapeutic approach to try and reduce gait asymmetry in individuals with neurological impairment.

## Materials and methods

### Participants

Fifteen healthy right-handed adults (five men and 10 women) aged 27 ± 7 years (mean ± SD), range 20–41, volunteered to participate in this study. As lateralization of the vestibular system depends on handedness (Dieterich et al., 2003), only right-handed participants were selected to decrease the interindividual variability and enable better interpretation of the results. The Edinburgh test of manual laterality was used to confirm handedness (Oldfield, 1971). Participants who reported a history of otologic, neurologic, cardiovascular, orthopedic, or traumatic illnesses were excluded.

### Experimental design

The experimental protocol was performed in two parts: first, the motor threshold (T) to the GVS was determined during standing, and then GVS was applied at an intensity of 1 or 1.5 T when participants walked at their comfortable speed on a treadmill.

### Instrumentation and evaluation

#### GVS

The vestibular system was evaluated in a binaural bipolar GVS configuration with a 200-ms pulse. First, 5-mm silver electrodes were placed on the mastoid process behind the ear. The skin on the mastoid process behind the ear was prepared with abrasive paper (3M Red Dot Trace Prep 2236, 3M Health Care, ON, Canada), and electrode cream was applied (EC2R Genuine Grass electrode cream 100 g, Natus, WI, USA). The electrodes were then fixed with tape (3M Transpore clear plastic tape, 2.5 cm, 3M Health Care). A cotton padding over the electrodes and a headband were used to ensure optimal contact between the electrode and the skin. A GRASS Electrode impedance meter EZM5C (Grass Technologies, RI, USA) was used to check the system impedance (≤1 KΩ at 30 Hz). A constant current stimulator was used to apply the GVS (Digitimer Ltd., DS22A, Cambridge, UK).

#### Accelerometer

The head is the first part of the body to tilt after GVS application (Day et al., 1997). We, therefore, measured the onset of head acceleration using a triaxial accelerometer positioned on the participant’s head to determine the motor threshold (MT) for the GVS. The participants wore tight, adjustable swimming caps. A triaxial accelerometer (46 g; Crossbow CXLOZLF3 ± 2 g Module) was placed on the vertex and attached to the cap using adhesive tape. The x-axis of the accelerometer was oriented with the tragus–tragus line, while the y-axis was aligned with the nasion and inion. The vertex of the head was determined as the intersection between both lines. The z-axis corresponded to the vertical with respect to gravity. The accelerometer sensitivity was set to 2 V/g.

#### EMG activity

The EMG signal of the right SOL was recorded using the Neurolog system (band-passed filtered: 10–1,000 Hz, gain: 100–1,000) with surface electrodes (AmbuR BlueSensor M, ECG Electrodes, Denmark) spaced 1.5–2.0-cm apart. After prepping the skin with abrasive tape, electrodes were placed on the right and left SOL and TA, which were selected due to their role in generating locomotor activity at the ankle (Fitzpatrick and Day, 2004). The electrodes were placed parallel to the muscle fibers and in accordance with SENIAM recommendations^1^ (Hermens et al., 2000). A reference electrode was positioned on the right tibial tuberosity.

### Determining the vestibular motor threshold

**Figure 1:**
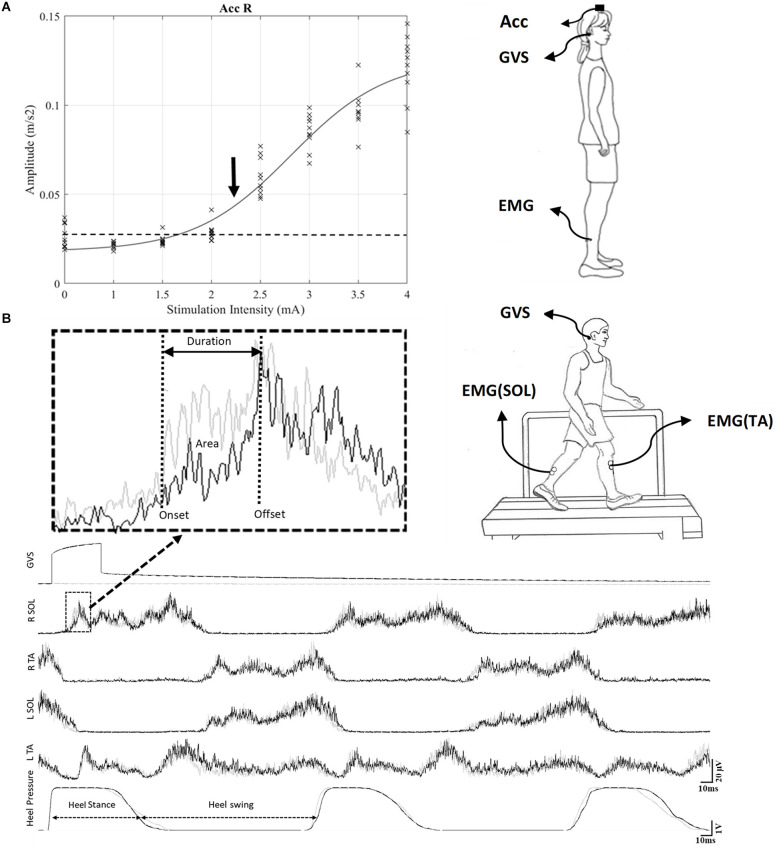
**(A)** Recruitment curve set-up during standing. To determine the intensity of stimulation, a recruitment curve was constructed to identify the motor threshold (T) to GVS (see Section “Methods”). The threshold was based on the signal from an accelerometer placed on top of the head (vertex) while subjects were standing with eyes closed. For this representative participant, the threshold was 2.3 mA. **(B)** GVS during gait on the treadmill set-up: The black trace shows the EMG pattern during the stimulated cycle, and the gray trace shows the EMG pattern during the control cycle. A typical GVS response is shown in the medallion and measures of onset, offset and area are indicated. GVS, galvanic vestibular stimulation; EMG, electromyography.

The complete procedure to determine the motor threshold to GVS is described in Mikhail et al. (2021), but briefly participants stood with their head facing forward, eyes closed, arms along the body, and wearing flat shoes on a force platform. The distance between the left and right medial malleoli was 50% of pelvic width, which was measured using calipers at the level of the greater trochanter. GVS was applied 10 times in random order for each of the following intensities: 0 (control), 1, 1.5, 2, 2.5, 3, 3.5, and 4 mA, with a minimum interval of 5 s between each stimulus. A recruitment curve was constructed using the response evoked by the GVS on the accelerometer signal. The intensity at which 50% of the responses exceeded the mean of background signal+1 SD was identified as an initial threshold (Figure 1A). Next, to obtain a more precise threshold, we stimulated at each 0.1 mA around the initial threshold to obtain a final threshold.

### GVS during gait

Participants were stimulated at 1 and 1.5 T (30 stimuli each) while walking on a treadmill at a comfortable speed (mean = 1.03 ± 0.06 m/s; Figure 1B). A pressure sensor was placed underneath the right heel to determine the gait cycle onset. The GVS was delivered at the onset of the stance phase of the right leg (10 ms after the pressure was increased under the right heel). Each participant took part in two sessions separated by at least 2 days. In one session, the cathode and anode were placed over the right and left mastoid process, respectively. In the other session, the electrode placement was reversed, with the cathode placed behind the left ear and the anode behind the right ear. The order of the sessions was randomized between the participants.

### Data analysis

#### EMG responses to GVS

Data from three participants had to be excluded due to technical errors during data collection, which made the data unusable for analysis. Therefore, the data of 12 participants were analyzed. Vestibular responses triggered by GVS in the right and left TA and SOL at the onset of the stance phase were rectified, averaged (*n* = 30 stimuli), and analyzed. Responses were considered SLR if their onset was between 50 and 90 ms; MLR, 90–150 ms; and LLR, 150–300 ms. The latency, duration, and amplitude of the response were determined (see Figure 1B). The onset of each response was identified as the first time point at which the EMG signal either fell below or rose above 1 standard deviation of the mean baseline activity observed during the control trials, for ≥10 ms. The offset corresponded to the first timepoint at which the signal returned to its mean baseline activity. Response duration was defined as the period between onset and offset. The area between the onset and offset of the response was measured to quantify response amplitude: the EMG area of the response was normalized to the mean EMG area over the same period in the control trials and then multiplied by 100. The response was either facilitatory (>100%) or inhibitory (<100%).

#### Cycle and EMG burst duration

The duration of a gait cycle represents the time between two successive contacts of the right heel. The mean cycle length of the stimulated gait cycle (STIM cycle), the following gait cycle (NEXT cycle), and a control cycle (CTRL cycle) were measured. To better understand the mechanisms underlying the changes in the gait cycle duration, the EMG duration of both SOL and TA were also measured. For each muscle, baseline activity was quantified by averaging the EMG level over 200 ms when no activity was detected in the muscle. The onset of the EMG burst was identified when the muscle activity exceeded the baseline EMG activity by two standard deviations for ≥30 ms. The end of the EMG burst was identified when the EMG activity decreased to the baseline EMG level.

### Statistical analysis

Data are expressed as means ± standard error of the mean. The Shapiro–Wilk test was used to test the normal distribution of the data. Two-way repeated-measures analysis of variance (ANOVA) was used to compare amplitude values of different configurations (RCathode vs. LCathode) and intensity (1 and 1.5 T). The effect of GVS on the cycle duration, heel stance duration, and EMG duration was assessed at 1 and 1.5 T using a one-way repeated-measures ANOVA. Tukey’s or Holm-Sidak multiple comparison tests were used to compare the control, stim, and next cycles. *T* test was used to investigate the effect of cathode configuration (right × left) on the response of each muscle to GVS at each intensity. *p* < 0.05 was considered statistically significant for all tests. We used Cohen’s d (Cd) to investigate effect size (small, ≤0.2; medium, 0.2–0.8; and large, ≥0.8; based on Lakens, 2013). All statistical analyses were conducted using the Prism software version 9.3.1 (GraphPad Software, Inc., San Diego, CA).

## Results

### Determination of stimulation intensity in the standing posture

Each participant took part in two sessions. In each session, a RCathode or a LCathode configuration was used. First, to determine the intensity of stimulation, a recruitment curve was performed for all participants, and the motor threshold (T) for vestibular response was identified, as illustrated in Figure 1A for a representative participant. For this participant, the threshold for RCathode was obtained at 2.3 mA. For the group, the mean thresholds were 2.6 ± 0.19 for RCathode and 2.06 ± 0.2 for LCathode. Next, two distinct intensities were used to evoke vestibular responses during gait: 1 and 1.5 T.

### Feasibility of GVS application during gait

GVS applied during gait did not induce any adverse events. However, strict guidelines were given with the participants prior to data collection. Notably, they had to always fix a point in front of them and refrain from turning their head to the side. Pilot testing indicated that when participants turned their head while stimulation was applied, they briefly became disoriented and had to hold on to the rails to prevent a fall. Because the participants were young and healthy, they could focus on the task, and no fall occurred. Therefore, the participants walked looking ahead and did not feel any discomfort. Some participants had tingling sensations and dry skin/itching behind their ears at the end of the session, as reported previously by others (Utz et al., 2011). However, these inconveniences were mild and brief.

### EMG responses evoked by GVS during ongoing locomotion

Each participant walked at their preferred speed on a treadmill (mean = 1.03 ± 0.06 m/s). Figure 1B shows the ongoing EMG pattern and heel strike during gait in a representative subject. The black traces represent the EMG activity when GVS is applied 10 ms after the onset of the gait cycle. It shows alternation between the TA and SOL in both the left and right legs, as well as the heel contact period (heel stance). The onset of the right heel contact determines the onset of the right stance phase and was referred to as the onset of the gait cycle. The gray trace shows the EMG and heel contact activity when no GVS was applied. The stimulated trace (black) is superimposed over the control trace (gray) and enables the identification of the responses evoked by GVS.

**Figure 2:**
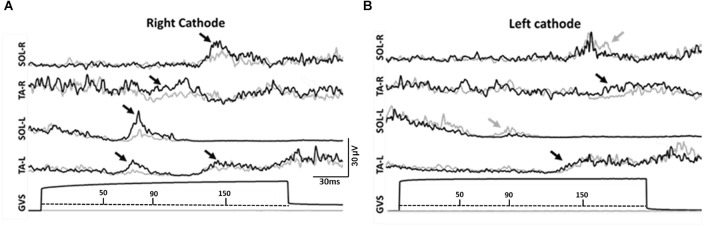
**(A)** Right cathode configuration. **(B)** Left cathode configuration. GVS stimulation during early stance produced SLR, MLR, and LLR in all muscles tested. Black arrows indicate facilitatory response, gray arrows indicate inhibitory response. 50 ms ≤ onset of Short Latency Response (SLR) ≥ 90 ms; 90 ms ≤ onset of Medium Latency Response (MLR) ≥ 150 ms; 150 ms ≤ onset of Long Latency Response (LLR).

Figures 2A,B details the response pattern observed in a representative participant for both RCathode and LCathode configurations. Although the responses varied within the group (see Supplementary Material), LLRs were the main responses observed for the right SOL, the right TA, and the left TA across intensities and configurations. Therefore, these were the main responses that were further assessed (Table 1). SLR was the main response observed in the left SOL and, accordingly, was further analyzed.

**Table 1:** EMG responses in left and right SOL and TA evoked by RCathode and LCathode configurations.

Right cathode							
Muscle	Main Response	Intensity					
		1 T			1.5 T		
		Latency (ms)	Duration (ms)	Amplitude (%control)	Latency (ms)	Duration (ms)	Amplitude (%control)
Right Soleus	LLR *	209.8 ± 14.2	39 ± 11.3	168.4 ± 13.4	184.1 ± 14.3	31.9 ± 11.6	202.4 ± 42.8
Right Tibialis Anterior	LLR *	189.6 ± 20.9	30.9 ± 4.5	201.5 ± 32.7	199.2 ± 25	63.6 ± 43.4	276 ± 66.9
Left Soleus	SLR **	63.8 ± 3.9	31.7 ± 8.5	189.2 ± 28.6	63.8 ± 4	47.5 ± 11.3	250.9 ± 53.7
Left Tibialis Anterior	LLR *	170.6 ± 16.1	21.6 ± 4.4	149.4 ± 8.2	179.7 ± 15.1	17 ± 3.6	147.7 ± 65.5
Left cathode							
Right Soleus	LLR *	197.4 ± 12.6	29.8 ± 5.8	91.7 ± 23.9	220.2 ± 3.4	29.2 ± 10.9	84 ± 14.2
Right Tibialis Anterior	LLR *	173.8 ± 9.6	33 ± 9.3	163.1 ± 29.6	184.2 ± 6.3	70.8 ± 30.1	174.9 ± 9.8
Left Soleus	SLR **	66.6 ± 7	46.8 ± 13.5	84.7 ± 29.8	64.5 ± 3.4	34.5 ± 8.5	92.3 ± 37.2
Left Tibialis Anterior	LLR *	187.8 ± 17.4	22.4 ± 3	69.5 ± 1.3	185.5 ± 9.3	29.3 ± 12.6	75.8 ± 1.6

*LLR, Long Latency Response; **SLR, Short Latency Responses.

### Responses in SOL

#### RCathode configuration

When stimulation was applied, the right leg was in early stance with EMG in the right SOL rapidly increasing and EMG in the left SOL rapidly decreasing (see Figure 1B). In the right SOL, LLR was the main response and consisted of the facilitation of the ongoing EMG both at 1 T (amplitude: 168.4% ± 13.4%; latency: 210 ± 10 ms; duration: 40 ± 10 ms) and at 1.5 T (amplitude: 202.4% ± 42.8%; latency: 180 ± 10 ms; duration: 30 ± 10 ms). In the left SOL, SLR comprised a facilitation of the ongoing EMG both at 1 T (amplitude: 189.2 ± 28.5%; latency: 60 ± 4 ms; duration: 30 ± 10 ms) and 1.5 T (amplitude: 250.9 ± 53.7%; latency: 60 ± 4 ms; duration: 50 ± 10 ms). Thus, mainly facilitatory responses were observed in SOL. No significant differences were observed in the amplitude, latency, or duration of responses between 1 and 1.5 T.

#### LCathode configuration

**Figure 3:**
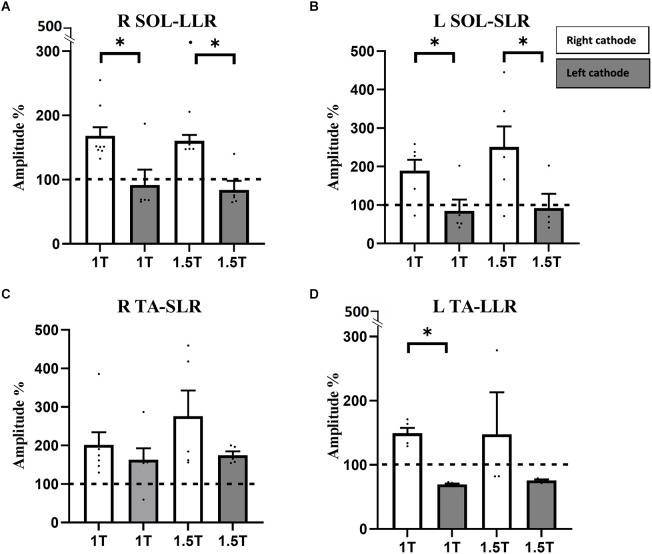
Responses in RSOL **(A)**, LSOL **(B)**, and L TA **(D)** were polarity dependent, but not statistically different between 1 and 1.5 T. Responses in R TA **(C)** were not polarity dependent. RSOL, right soleus; LSOL, left soleus; L TA, left tibialis anterior muscles; R TA, tibialis anterior muscles. Statistical significance is shown with an asterisk (*).

In the right SOL, LLR consisted of suppression of the ongoing right SOL EMG at 1 T (amplitude: 91.7 ± 23.9%; latency: 200 ± 10 ms; duration: 30 ± 10 ms) and 1.5 T (amplitude: 83.97 ± 14.19%; latency: 220 ± 3 ms; duration: 30 ± 10 ms). In the left SOL, SLR also comprised a suppression of ongoing EMG at 1 T (amplitude: 84.8 ± 29.9%; latency: 70 ± 10 ms; duration: 50 ± 10 ms) and 1.5 T (amplitude: 92.3 ± 37.2%; latency: 60 ± 3 ms; duration: 30 ± 10 ms). No significant differences were observed in the amplitude, latency, and duration of responses between 1 and 1.5 T in either SOL. Therefore, responses in SOL are polarity dependent: they facilitate ongoing EMG in the RCathode configuration and suppress ongoing EMG in the LCathode configuration (Figures 3A,B). This polarity-dependency is significant, as differences in amplitudes between the configurations are significant both at 1 T (*p*_the right SOL_ = 0.042, Cd = 1.62, *p*_the left SOL_ = 0.0328, Cd = 1.52) and 1.5 T (*p*_the right SOL_ = 0.0025, Cd = 1.42, *p*_the left SOL_ = 0.0416, Cd = 1.48).

### Responses in TA

#### RCathode configuration

A larger variety of responses were observed in TA compared to SOL (see Supplementary Material). In right TA, facilitatory LLRs were observed at 1 T (amplitude: 201.5 ± 32.7%, latency: 190 ± 20 ms, duration: 30 ± 4 ms) and 1.5 T (amplitude: 275.96 ± 66.94%, latency: 200 ± 30 ms, duration: 60 ± 40 ms; Figure 3C). In the left TA, facilitatory LLR were observed at 1 T (amplitude: 149.4 ± 8.2%, latency: 170 ± 20 ms, duration: 20 ± 5 ms; Figure 3D) and at 1.5 T (amplitude: 147.7 ± 65.5%, latency: 200 ± 10 ms, duration: 20 ± 4 ms). No significant difference was observed in the amplitude, latency, and duration of responses observed at 1 and 1.5 T.

#### LCathode configuration

In the right TA, responses facilitated ongoing EMG at 1 T (amplitude: 163.1 ± 29.6%, latency: 170 ± 10 ms, duration: 30 ± 10 ms) and at 1.5 T (amplitude: 174.9 ± 9.8%, latency: 180 ± 10 ms, duration: 70 ± 30 ms; Figure 3C). In the left TA, LLRs were inhibitory at 1 T (amplitude: 69.5 ± 1.3%, latency: 200 ± 20 ms, duration: 200 ± 3 ms) and 1.5 T (amplitude: 75.8 ± 1.6%, latency: 200 ± 10 ms, duration: 30 ± 10 ms; Figure 3D). No significant differences were observed in the latency and duration of responses between 1 and 1.5 T in either TA. Whereas no difference was observed in amplitude for the right TA, the amplitude of the response was larger at 1.5 T than at 1 T in the left TA (*p* = 0.0194, Cd = 2.12). Overall, in the right TA, LLRs were facilitatory regardless of the polarity, and no significant difference was observed between responses obtained with RCathode and LCathode, either at 1 T (*p* = 0.402, Cd = 0.48) or 1.5 T (*p* = 0.2066, Cd = 0.94; Figure 3C). In the left TA, the nature of the LLR depended on the polarity, and a significant difference was observed in the amplitude of the responses obtained between RCathode and LCathode at 1 T (*p* < 0.0001, Cd = 6.1) but was marginally significant at 1.5 T (*p* = 0.0571, Cd = 0.89; Figure 3D).

### Effect of GVS on gait cycle duration

#### RCathode configuration

**Figure 4:**
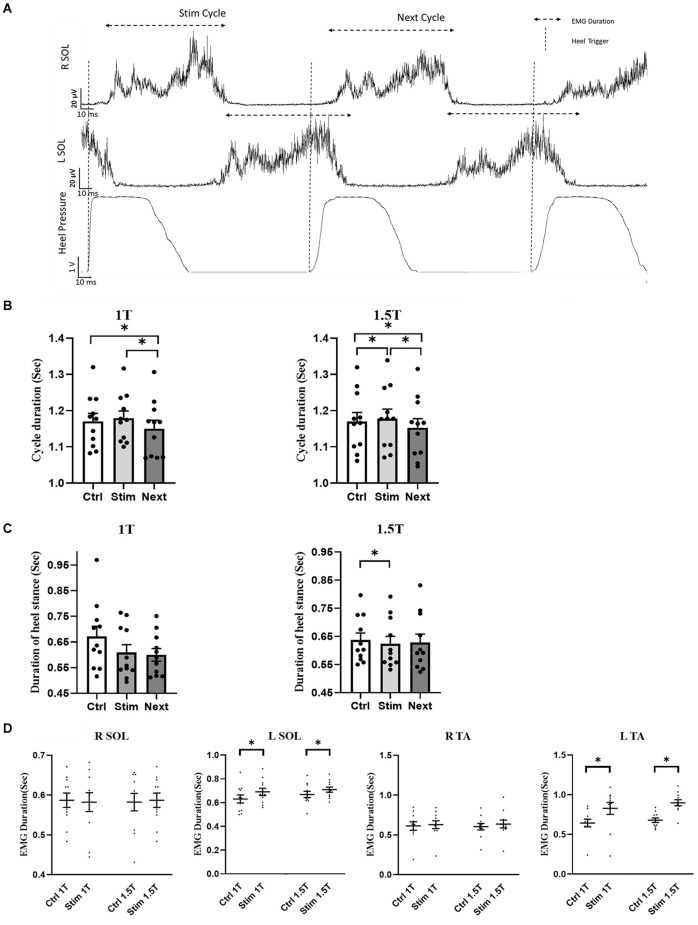
R Cathode Configuration. **(A)** Averaged and rectified EMG signal of left and right soleus (SOL) muscles during locomotion. The gait cycle duration was measured during stimulation (Stim cycle) and also for the cycle following the stimulation (Next cycle), as well as in a control cycle (not shown). **(B)** The group data show that the duration of the Stim cycle increases significantly during stimulation at 1.5 T, but not at 1 T. Furthermore, next cycle duration is significantly decreased compared to the control cycle, and the Stim cycle at both 1 T and 1.5 T. **(C)** Duration of the heel stance was decreased in the stim cycle compared to control, which was statistically significant only at 1.5 T. No significant difference was detected for the Next cycle. **(D)** The duration of the EMG burst in LSOL, and LTA was increased in the Stim cycle compared to control at both 1 T and 1.5 T. No significant difference was observed in the duration of RTA and RSOL EMG burst during Stim Cycle. Statistical significance is shown with an asterisk (*).

Figure 4 summarizes the influence of GVS on cycle duration in the RCathode configuration by comparing cycle length between the control, stimulated, and next cycles (Figure 4A). GVS increased the duration of the stimulated cycle compared with the duration of the control cycle at 1.5 T (*p* = 0.0312, Cd = 0.094). However, this increase was not significant at 1 T (*p* = 0.234, Cd = 0.12; Figure 4B). The duration of the cycle that follows the stimulated cycle, which is referred to as the NEXT cycle, was decreased compared with the control cycle at both 1 T (*p* = 0.001, Cd = 0.27) and 1.5 T (*p* = 0.0017, Cd = 0.21). The stimulated cycle was also always longer than the next cycle at both intensities (*p*_1T_ < 0.001, Cd = 0.4 and *p*_1.5T_ < 0.0001, Cd = 0.3). While the cycle duration was increased in the STIM cycle, the time the participants spent in stance on the right heel (right heel pressure) was decreased. Figure 4C shows a decrease in heel stance at 1 T, which is significant at 1.5 T (*p* = 0.0447, Cd = 0.16). To better understand the changes underlying the lengthening of the STIM cycle, the EMG duration of ankle muscles were analyzed further (see Figure 4A). No significant changes were observed in the EMG duration of the right SOL or TA at 1 T or 1.5 T. However, the duration of the EMG burst in the left TA and the left SOL increased in the stim cycle compared with the control cycle both at 1 T (left TA: 830 ± 80 vs. 640 ± 50 ms, *p* = 0.035, Cd = 0.9; left SOL: 690 ± 30 vs. 630 ± 30 ms, *p* = 0.008, Cd = 0.56) and at 1.5 T (TA: 900 ± 40 vs. 680 ± 30 ms, *p* = 0.002, Cd = 1.99; left SOL: 710 ± 20 vs. 670 ± 30 ms, *p* = 0.004, Cd = 0.5; Figure 4D).

#### LCathode configuration

**Figure 5:**
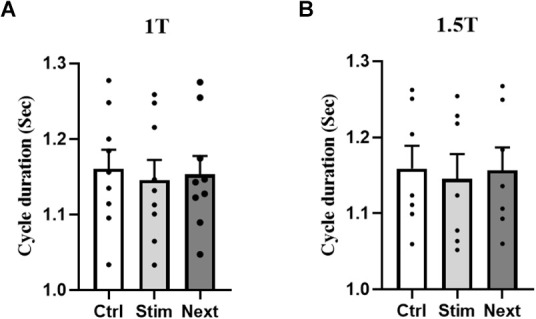
L Cathode Configuration. No statistical difference in cycle duration was observed between the control, stim, or next cycles at 1 T **(A)** and 1.5 T **(B)**.

Whereas GVS increases STIM cycle duration in the RCathode configuration, there was no significant difference in the duration of the gait cycle when GVS was applied in a LCathode configuration (*p*_1T_ = 0.136 and *p*_1.5T_ = 0.191; Figure 5).

## Discussion

In this study, we applied a short-duration binaural, bipolar vestibular stimulation at the onset of the gait cycle of the right leg to characterize vestibular responses and determine the effects of GVS on cycle duration during walking in healthy participants. Responses evoked in the right SOL, the left SOL, and the left TA were polarity dependent, being facilitatory in a RCathode configuration and inhibitory in a LCathode configuration. Responses triggered in the right TA were facilitatory regardless of polarity. Furthermore, GVS applied in a RCathode configuration increased the duration of the stimulated cycle compared with the duration of the control cycle. This lengthening was due to the increased duration of the EMG bursts in the left TA and the left SOL compared with the control at both 1 and 1.5 T. No change in EMG burst duration was observed in right TA and SOL. Overall, R Cathode GVS prolonged the stimulated gait cycle, and these effects were mediated through increased EMG burst duration on the anodic side.

### Characteristics of the vestibular responses during gait vs. standing

Responses were facilitatory in both left and right SOL in an RCathode configuration, and they were inhibitory in both SOL in an LCathode configuration. Observing the same responses in both legs during gait is different from what would be expected by applying bipolar, binaural GVS while standing with the head facing forward. In the latter condition, responses between the left and right SOL are generally reversed with the opposite polarity (Lund and Broberg, 1983; Pastor et al., 1993; Fitzpatrick and Day, 2004). Observing similar responses on both sides was previously observed during gait, which suggests it might be inherent to the locomotor task (Iles et al., 2007). Except for the left SOL, most responses observed during gait were LLRs, which again contrasts with responses evoked during standing, with the MLR being the most analyzed. This finding suggests that longer latency responses may be a feature of vestibular responses during gait, as was proposed previously (Iles et al., 2007).

While LLRs were observed in the RSOL, only SLRs were observed in the LSOL. One explanation might be that L SOL EMG rapidly decreases at the offset of stance to give way to swing and to TA contraction (Simonsen, 2014). As EMG activity is absent during swing, it might not be possible to see a response at later latencies.

The nature of the dominant vestibular responses (SLR, MLR, and LLR) in the TA was not as clear as in the SOL, and a larger variability was observed depending on the intensity and GVS configuration. The apparent stability in the responses observed in SOL could reflect the preferred connectivity of vestibulospinal neurons to extensor muscles during gait, as was shown for treadmill walking in cats (Orlovsky, 1972; Matsuyama and Drew, 2000). Furthermore, as we are stimulating at the onset of the stance of the right leg, we are in the double support phase where balance is more of a challenge (Nielsen, 2003; Simonsen, 2014). The weight transfer implies coordination between the right SOL EMG, which is rapidly increasing to accept the body weight at the onset of stance, and the left SOL EMG, which is also increasing for push off at the offset of stance and then quickly decreasing to start the swing phase. This could explain the more robust and repeatable responses obtained in SOL, as clear responses are mainly observed in muscles actively engaged in a balance task (Wardman et al., 2003; Fitzpatrick and Day, 2004).

### Significant effect of RCathode GVS on gait cycle duration

RCathode GVS prolonged the duration of the ongoing gait cycle by increasing the burst duration of muscles located on the anodic side. This more important effect on the anodal side echoes previous findings: when applying GVS while standing, the main behavioral response is a deviation of the body (lean) toward the anode (Britton et al., 1993; Fitzpatrick and Day, 2004). Furthermore, the GVS also caused participants walking overground to deviate their trajectory toward the anode (Bent et al., 2000). Although the effect of GVS on EMG burst duration was not assessed in the latter study, the stimulation might have prolonged the EMG on the anodic side and contributed to the deviation. Moreover, the RCathode GVS also influenced the subsequent gait cycle and shortened its duration. This may reflect a compensatory strategy aimed at recovering a normal length cycle during gait (Eng et al., 1994; Schillings et al., 2000; Nachmani et al., 2020). The involvement of brainstem networks in determining burst and cycle duration was also observed during fictive locomotion in neonatal mice. Removal of the brainstem decreases the burst duration (Jean-Xavier and Perreault, 2018). In parallel, in patients with chronic bilateral vestibular loss gait initiation is characterized by a lower maximum displacement of the center of pressure n the first- and second-steps compared with control participants (Sasaki et al., 2001).

In the current study, RCathode GVS induces prolonged burst duration on the anodic side, which might be explained by altered perception/sensation induced by GVS. Bilateral bipolar transmastoid GVS causes a whole body sway directed toward the ear with the anode, irrespective of the orientation of the head in the yaw plane (Pastor et al., 1993). Fitzpatrick and Day (2004) introduced a vector summation model, suggesting that the brain interprets bilateral bipolar GVS predominantly as a “rotation toward the side of the cathode,” and that in reaction to this perception, the evoked postural response (body sway) is directed toward the anode. Hence, participants would perceive that they were falling toward the cathodic side, i.e., on the right as they are putting their right foot on the ground. As a reaction, the participant would have a postural response toward the left side (anode), in the subsequent step. This reaction could explain the longer EMG bursts on the anodic side (left), while the cathode was behind the right ear. The opposite GVS configuration (LCathode-RAnode) would not be in congruence with the postural reaction during the stimulated cycle, because the participant will perform a step with the left leg following stimulation, which is on the cathode side.

An alternative explanation for the difference in the effects triggered by the left and right cathode is the vestibular dominance of the right-handed participants recruited. As the vestibular system is lateralized (Dieterich et al., 2003), the right cathode might have a more preponderant effect on the vestibular system in the right-handed participants. Although this is only a hypothesis, it rests on previous data (Fink et al., 2003) showing that the right and left cathode stimulation activated different parts of the brain in right-handed participants and that different regions are activated by GVS in right- and left-handers (Kirsch et al., 2018). A similar asymmetry could occur caudally in vestibular projections to the spinal cord, which would lead to more dominant responses when the cathode is on the right side. Further studies could shed light on this hypothesis.

### Brainstem as a relay station of vestibular afferents in the control of gait

Behavioral responses observed during GVS are due to the activation of the peripheral vestibular system. GVS was found to predominantly activate or inhibit thick irregularly firing afferent fibers from both semicircular canals and otoliths (Goldberg et al., 1982, 1984), as well as hair cells (Norris et al., 1998; De Waele et al., 2002; Cheng et al., 2010; Gensberger et al., 2016). Cathodal current increases action potential discharge, whereas anodal currents silence vestibular afferents (Angelaki and Perachio, 1993; Straka and Dieringer, 2000). The different afferent signals will then be processed centrally, weighted, and integrated with other sensory inputs. The information would be transmitted to the lower limb either directly through the vestibulospinal tract or indirectly through the reticulospinal tract (Wilson et al., 1979; Kennedy et al., 2004). These descending pathways then modulate MN excitability through polysynaptic or disynaptic interneuronal networks (Gossard et al., 1996). Therefore, segmental interneurons that regulate MN excitability and are activated or inhibited by changes in vestibular afferent discharge could contribute to these SLR, MLR, and LLR responses. Notably, Renshaw cells provide recurrent inhibitory input to MNs and respond to descending vestibulospinal input (Pompeiano, 1988). Increased activity of vestibular afferents (following cathodal stimulation) could facilitate the motoneuron pool by inhibiting Renshaw cells (Kennedy et al., 2004). Another potential mechanism is the modulation of reciprocal inhibition and presynaptic inhibition interneurons through both vestibulospinal and reticulospinal pathways (Manzoni, 1988; Iles and Pisini, 1992; Kennedy et al., 2004).

Vestibular signals can also be integrated into the ongoing gait pattern. The Central pattern generator (CPG) enables a specific pattern of muscle activation that leads to flexion/extension and left–right alternation (Rossignol et al., 2006). It also adapts the locomotion to incoming sensory feedback (notably from the treadmill belt). This intrinsic spinal locomotor network might have a gating effect on the potential modulation of locomotion by vestibular afferents (Guillaud et al., 2020). This possibility might underlie the small effect size we observed in the prolonged duration of the gait cycle, especially when compared to the larger effect size of vestibular responses induced in the muscles (SLR, MLR, and LLR). As vestibular signal relays information about head movement, such gating might be used to reduce the influence of head movement on the locomotor pattern, as suggested by others (Pflieger and Dubuc, 2004).

### Limitations

A limitation of this study is that the gait cycle was only assessed using heel sensors on one side (the right side); thus, the duration of the gait cycle itself was not assessed on both legs, which prevents direct assessment of symmetry. Moreover, the participants were tested only with eyes open and at comfortable walking speeds. As the modulation of EMG by GVS is larger with eyes closed and at slower speed, a wider bracket of speeds should be tested, and both eyes open and eyes closed conditions should be investigated.

Only right-handed individuals were tested, which prevents extrapolation of our conclusion. Future studies should include both right-handed and left-handed individuals. Moreover, our cohort was young, and the results might be different in older adults, where GVS sensitivity is generally increased (Jahn et al., 2003; Dalton et al., 2014; Peters et al., 2016). Furthermore, although the lengthening of muscle activity on the anodal side underlies the lengthening of cycle duration, the data do not inform on the specific contribution of the vestibular system to the muscular changes observed. In subsequent steps, other techniques such as time and frequency correlation approaches (see Blouin et al., 2011) should be used to clarify the vestibulo-muscular coupling during the gait cycle.

Another limitation is that the recruitment curve was taken during standing and not during gait. Although the latter approach would have enabled a better approximation of the excitability of the neuronal networks during gait, it was technically more of a challenge due to time constraints. Indeed, a large amount of stimulation was necessary to construct this curve. During standing, we can apply this stimulation relatively quickly in a randomized manner. During gait, it takes longer, as we do not want the stimulation to be applied in consecutive cycles, and we offered frequent resting periods to participants to prevent fatigue.

### Potential clinical impact

The main effect of a short-duration GVS burst applied at the onset of the right gait cycle was the prolongation of EMG activity on the left side (anodic side) without changing EMG activation on the right side. Repeating this stimulation at each cycle might enable the lengthening of the gait cycle over the course of a sequence of locomotion. This approach might be useful in a therapeutic/training paradigm with people with asymmetrical gait, for example, after a stroke (Verma et al., 2012). Indeed, asymmetrical gait is an important issue in the rehabilitation of patients with neurological disorders, and one that has received much attention is poststroke gait impairment (Patterson et al., 2010; Verma et al., 2012; Beyaert et al., 2015). Gait asymmetry is reflected by a significant difference in the length of the left and right gait cycles (Kim and Eng, 2003; Patterson et al., 2010; Wonsetler and Bowden, 2017). During therapeutic interventions, the restoration of symmetrical gait (Harris-Love et al., 2001; Lindquist et al., 2007; Kahn and Hornby, 2009) is an important goal, but it is rarely fully achieved. Studies have demonstrated that asymmetrical gait can be reduced in patients with stroke by using asymmetrical somatosensory cues, such as those provided by a split-belt treadmill (Reisman et al., 2007; Malone and Bastian, 2014). Our results suggest that asymmetrical vestibular cues might effectively address gait asymmetry as well. With a configuration where the cathode is on the nonparetic side, repeatedly applying a low-intensity GVS burst at the onset of the gait cycle could lengthen EMG bursts on the paretic side and increase cycle duration. Future studies should also determine if a short burst, a longer burst, or continuous GVS (such as nGVS) with an RCathode configuration might be appropriate in that regard.

## Conclusion

In conclusion, this study demonstrates that a brief, low-intensity GVS pulse delivered at the onset of stance can increase the duration of the gait cycle by prolonging EMG activity of ankle muscles located on the anode side, but not on the cathode side. A similar approach could be explored to address deficits in gait symmetry in individuals with neurological impairments.

## Data availability statement

The original contributions presented in the study are included in the article/Supplementary material, further inquiries can be directed to the corresponding author.

## Ethics statement

This study involved human participants and was approved by the Research Ethics Board of the Center for Interdisciplinary Research in Rehabilitation of Greater Montreal. It was conducted in accordance with the principles established in the Declaration of Helsinki. The participants provided their written informed consent to participate in this study.

## Author contributions

YM and DB conceived and designed research. YM, JC, and DB performed experiments. FA, AH, and DB analyzed data and prepared figures. FA, AH, YM, J-MM-T, and DB interpreted results of experiments. FA drafted manuscript. FA, AH, YM, JC, J-MM-T, and DB edited and revised manuscript. DB approved final version of manuscript. All authors contributed to the article and approved the submitted version.

## Footnotes

^1^ seniam.org
