# Supplementary material for: Effect of galvanic vestibular stimulation applied at the onset of stance on muscular activity and gait cycle duration in healthy individuals
Source: Front Neural Circuits. 2023 Feb 9;16:1065647. doi: 10.3389/fncir.2022.1065647 (PMC9946991; doi:10.3389/fncir.2022.1065647)
Supplement: Supplementary file 1 [file Table_1.pdf]

Supplementary Material

Effect of galvanic vestibular stimulation applied at the onset of stance on muscle responses and gait cycle duration in healthy individuals.

Faezeh Abbariki, Youstina Mikhail, Adjia Hamadjida, Jonathan Charron, Jean-Marc Mac-Thiong, and Dorothy Barthélemy.

*** Correspondence:**

Dorothy Barthélemy, PhD, MSc, PT

School of Rehabilitation, Faculty of Medicine

Université de Montréal

Pavillon du Parc,

C.P.6128 Succ. Centre-ville

Montreal Qc, Canada, H3C 3J7

Phone: (514) 343-7712 Fax: (514) 343-6929

E-mail: dorothy.barthelemy@umontreal.ca

Supplementary Tables

Right Cathode Configuration - 1T									
	R SOL								
	SLR			MLR			LLR		
	Latency (ms)	Duration (ms)	Amplitude (%control)	Latency (ms)	Duration (ms)	Amplitude (%control)	Latency (ms)	Duration (ms)	Amplitude (%control)
**PP 001**				148	45	141.63	267	35	151.89
**PP 003**	55	46	53.43	106	19	60.19	189	13	167.14
**pp 004**				116	14	166.76	185	24	254.83
**pp 007**							278	127	215.29
**pp 008**							168	34	150.83
**pp 009**							155	34	144.89
**pp010**	71	18	67.98	112	22	62.65	227	27	151.48
**pp 013**							195	37	146.36
**pp014**				96	23	58.02	224	20	132.72
**Mean**	63	32	60.71	115.6	24.6	97.85	209.8	39	168.38
**SEM**	8	14	7.28	8.8	5.4	23.35	14.2	11.3	13.36

Right Cathode Configuration -1T									
ID	L SOL								
	SLR			MLR			LLR		
	Latency (ms)	Duration (ms)	Amplitude (%control)	Latency (ms)	Duration (ms)	Amplitude (%control)	Latency (ms)	Duration (ms)	Amplitude (%control)
**PP 001**	67	62	258.40						
**PP 003**	66	49	238.10						
**pp 004**	56	15	227.86				164	19	58.95
**pp 005**	77	35	195.80						
**pp 007**	67	8	142.18						
**pp 008**	50	21	72.91	103	29	139.56			
**pp010**				121	16	137.07			
**pp 013**				93	52	225.83			
**Mean**	63.8	31.7	189.21	105.7	32.3	167.49	164	19	58.95
**SEM**	3.9	8.5	28.55	8.2	10.5	29.18			

Right Cathode Configuration -1T									
ID	R TA								
	SLR			MLR			LLR		
	Latency (ms)	Duration (ms)	Amplitude (%control)	Latency (ms)	Duration (ms)	Amplitude (%control)	Latency (ms)	Duration (ms)	Amplitude (%control)
**PP 001**	87	58	149.11				234	23	146.74
**pp 004**	51	36	73.62				184	50	222.22
**pp 005**				111	59	184.60			
**pp 008**	66	22	46.18	139	28	46.83	193	28	190.63
**pp 009**	62	22	73.26				153	18	129.74
**pp010**							98	40	161.56
**pp 013**	71	33	64.04	139	108	45.66	271	20	385.56
**pp014**	80	35	59.54				194	37	174.39
**Mean**	69.5	34.3	77.63	129.7	92.4	92.36	189.6	30.9	201.55
**SEM**	5.3	5.9	14.88	9.3	23.3	46.12	20.9	4.5	32.70

Right Cathode Configuration -1T									
ID	L TA								
	SLR			MLR			LLR		
	Latency (ms)	Duration (ms)	Amplitude (%control)	Latency (ms)	Duration (ms)	Amplitude (%control)	Latency (ms)	Duration (ms)	Amplitude (%control)
**PP 001**	65	50	202.74	147	19	146.86			
**PP 003**				125	22	135.39			
**pp 004**				107	14	143.65	184	39	171.03
**pp 005**	83	20	190.09				223	18	149.24
**pp 007**	65	10	157.00						
**pp 008**	85	30	60.10	115	31	150.82	147	17	133.75
**pp 009**				136	25	138.04			
**pp 013**				93	31	184.71	170	19	129.03
**pp014**							129	15	163.97
**Mean**	74.5	27.5	152.48	120.5	23.7	149.91	170.6	21.6	149.40
**SEM**	5.5	8.5	32.27	8.02	2.7	7.33	16.1	4.4	8.19

Right Cathode Configuration -1.5T									
ID	R SOL								
	SLR			MLR			LLR		
	Latency (ms)	Duration (ms)	Amplitude (%control)	Latency (ms)	Duration (ms)	Amplitude (%control)	Latency (ms)	Duration (ms)	Amplitude (%control)
**PP 001**							185	19	147.25
**PP 003**	72	40	59.29	137	27	148.17			
**pp 005**							193	25	154.64
**pp 007**	87	24	65.01	137	17	56.59	261	100	205.59
**pp 009**							166	31	158.79
**pp010**				0.123	29	60.75			
**pp 013**							148	14	454.81
**pp014**	77	20	70.06				154	17	147.90
**PP015**				97	34	54.58	182	17	147.95
**Mean**	78.7	28	64.79	92.8	26.7	80.02	184.1	31.9	202.42
**SEM**	4.4	6.1	3.11	32.3	3.6	22.75	14.3	11.6	42.78

Right Cathode Configuration -1.5T									
ID	L SOL								
	SLR			MLR			LLR		
	Latency (ms)	Duration (ms)	Amplitude (%control)	Latency (ms)	Duration (ms)	Amplitude (%control)	Latency (ms)	Duration (ms)	Amplitude (%control)
**PP 001**	75	60	344.03						
**PP 003**	47	72	224.83						
**pp 004**	60	14	71.31						
**pp 005**	68	65	253.17						
**pp 008**				95	21	145.93			
**pp 009**				109	15	143.93			
**pp 013**	70	64	445.24						
**pp015**	63	10	166.67						
**Mean**	63.8	47.5	250.88	102	18	144.93			
**SEM**	4	11.3	53.69	7	3	1			

Right Cathode Configuration -1.5T									
ID	R TA								
	SLR			MLR			LLR		
	Latency (ms)	Duration (ms)	Amplitude (%control)	Latency (ms)	Duration (ms)	Amplitude (%control)	Latency (ms)	Duration (ms)	Amplitude (%control)
**PP 001**				90	47	173.33			
**pp 004**				107	14	130.83			
**pp 005**				101	39	165.54			
**pp 007**				113	17	139.66	280	22	184.25
**pp 008**				90	48	192.15	143	237	417.97
**pp 009**									
**pp010**				110	30	194.57	160	28	162.05
**pp 013**				143	20	454.39	182	13	459.48
**pp014**							231	18	156.02
**pp015**	75	29	156.8						
**Mean**	75	29	156.8	107.7	30.7	207.21	199.2	63.6	275.95
**SEM**				6.8	5.4	42.19	25.03	43.42	66.94

Right Cathode Configuration -1.5T									
ID	L TA								
	SLR			MLR			LLR		
	Latency (ms)	Duration (ms)	Amplitude (%control)	Latency (ms)	Duration (ms)	Amplitude (%control)	Latency (ms)	Duration (ms)	Amplitude (%control)
**PP 001**	73	29	176.52						
**pp 004**	70	10	154.04				151	10	82.08
**pp 005**	79	29	154.09						
**pp 007**	79	12	159.67						
**pp010**	81	34	63.48				186	19	82.31
**pp 013**				141	20	454.08	202	22	278.71
**pp014**	67	11	64.25	87	17	127.64			
**pp015**				137	10	74.72			
**Mean**	74.8	20.8	128.68	121.7	15.7	218.81	179.7	17	147.70
**SEM**	2.3	4.5	20.77	17.4	3	118.62	15.1	3.6	65.51

Left Cathode Configuration -1T									
ID	R SOL								
	SLR			MLR			LLR		
	Latency (ms)	Duration (ms)	Amplitude (%control)	Latency (ms)	Duration (ms)	Amplitude (%control)	Latency (ms)	Duration (ms)	Amplitude (%control)
PP 001							166	16	68.79
PP 003				103	31	65.23	170	49	187.24
pp 007	68	54	184.80						
pp 012	85	13	64.88				218	20	68.69
pp 013	76	76	171.81						
pp 014							205	31	68.21
pp015				127	12	155.56	228	33	65.62
Mean	76.3	47.7	140.50	115	21.5	110.4	197.4	29.8	91.71
SEM	4.9	18.5	37.99	12	9.5	45.17	12.6	5.8	23.89

Left Cathode Configuration -1T									
ID	L SOL								
	SLR			MLR			LLR		
	Latency (ms)	Duration (ms)	Amplitude (%control)	Latency (ms)	Duration (ms)	Amplitude (%control)	Latency (ms)	Duration (ms)	Amplitude (%control)
PP 001	76	47	41.68						
Pp 004				109	31	128.34			
PP 003	81	10	73.90	98	29	229.57			
pp005	45	93	52.24						
pp 007	55	49	202.28						
pp 013	76	35	53.53						
Mean	66.6	46.8	84.73	103.5	30	178.96			
SEM	7	13.5	29.85	5.5	1	50.62			

Left Cathode Configuration -1T									
ID	R TA								
	SLR			MLR			LLR		
	Latency (ms)	Duration (ms)	Amplitude (%control)	Latency (ms)	Duration (ms)	Amplitude (%control)	Latency (ms)	Duration (ms)	Amplitude (%control)
**PP 001**	49	20	136.06	93	21	77.74	160	69	155.84
**Pp 004**				148	35	161.09			
**pp 007**	86	30	140.60				219	15	164.38
**pp 012**	89	15	201.21	116	43	239.09	178	51	286.97
**pp 013**	63	15	124.36	111	43	151.73	169	31	157.84
**pp 014**				111	27	151.20	162	11	59.17
**PP 015**							155	21	154.38
**Mean**	71.8	20	150.56	115.8	33.8	156.17	173.8	33	163.10
**SEM**	9.6	3.5	17.23	9	4.4	25.59	9.6	9.3	29.61

Left Cathode Configuration -1T									
ID	L TA								
	SLR			MLR			LLR		
	Latency (ms)	Duration (ms)	Amplitude (%control)	Latency (ms)	Duration (ms)	Amplitude (%control)	Latency (ms)	Duration (ms)	Amplitude (%control)
**PP 001**				135	23	171.56	235	19	67.15
**PP 003**				110	33	64.08			
**pp005**				121	31	155.75	226	19	73.02
**pp 007**	63	29	117.16				155	34	71.98
**pp 012**	73	17	142.12	140	11	164.60	160	18	68.77
**pp 013**				105	37	177.42	163	22	66.79
**pp 014**	60	17	66.55						
**PP 015**	84	16	75.32						
**Mean**	70.	19.75	100.29	122.2	27	146.68	187.8	22.4	69.54
**SEM**	5.43	3.6	20.54	6.8	4.6	20.96	17.4	3	1.26

Left Cathode Configuration -1.5 T									
ID	R SOL								
	SLR			MLR			LLR		
	Latency (ms)	Duration (ms)	Amplitude (%control)	Latency (ms)	Duration (ms)	Amplitude (%control)	Latency (ms)	Duration (ms)	Amplitude (%control)
PP 003	69	53	49.96	141	37	139.67	218	10	140.19
pp005				120	34	141.08	211	70	66.73
pp 007				125	74	162.79	230	14	64.78
pp 013	65	44	150.86	119	29	173.10	226	33	75.50
pp 014							216	19	72.65
PP 015	80	68	147.35						
Mean	71.3	55	116.06	0.13	0.04	154.16	220.2	29.2	83.97
SEM	4.5	7	33.06	0.01	0.01	8.24	3.4	10.9	14.19

Left Cathode Configuration -1.5T									
ID	L SOL								
	SLR			MLR			LLR		
	Latency (ms)	Duration (ms)	Amplitude (%control)	Latency (ms)	Duration (ms)	Amplitude (%control)	Latency (ms)	Duration (ms)	Amplitude (%control)
PP 003	74	52	202.50						
pp005				131	50	156.55	265	42	177.07
pp 013	64	46	41.38	130	17	133.75			
pp 014	62	21	69.78						
PP 015	58	19	55.53						
Mean	64.5	34.5	92.30	130.5	33.5	145.15	265	42	177.07
SEM	3.4	8.5	37.19	0.5	16.5	11.40			

Left Cathode Configuration - 1.5T									
ID	R TA								
	SLR			MLR			LLR		
	Latency (ms)	Duration (ms)	Amplitude (%control)	Latency (ms)	Duration (ms)	Amplitude (%control)	Latency (ms)	Duration (ms)	Amplitude (%control)
**PP 001**				107	46	72.63	183	148	200.10
**pp 004**									
**pp005**	86	13	100	143	27	70.19	192	141	196.55
**pp 007**							202	24	157.43
**pp 013**	82	17	71.39	104	125	183.03			
**pp 014**							164	21	165.71
**PP 015**	75	11	62.93	109	61	179.64	180	20	154.57
**Mean**	81	13.7	67.16	115.8	64.8	126.37	184.2	70.8	174.87
**SEM**	3.2	1.8	4.23	9.1	21.3	31.74	6.3	30.1	9.76

Left Cathode Configuration - 1.5T									
ID	L TA								
	SLR			MLR			LLR		
	Latency (ms)	Duration (ms)	Amplitude (%control)	Latency (ms)	Duration (ms)	Amplitude (%control)	Latency (ms)	Duration (ms)	Amplitude (%control)
**PP 001**				0.141	0.022	175.45			
**pp005**				0.099	0.063	184.50	181	66	71.57
**pp 007**	84	27	153.323	0.116	0.014	70.26			
**pp 013**	50	32	66.6547	0.103	0.038	218.22	174	25	76.47
**pp 014**				0.120	0.011	79.58	174	14	79.10
**PP 015**	75	21	59.1867	0.118	0.011	76.75	213	12	76.22
**Mean**	69.7	26.7	93.05	0.12	0.03	134.13	185.5	29.3	75.84
**SEM**	10.2	3.2	30.21	0.01	0.01	26.87	9.3	12.6	1.57

Right Cathode Configuration -1T			
Cycle duration (ms)			
ID	Ctrl	Stim	Next
PP001	1320	1317	1306
PP003	1184	1177	1171
PP004	1144	1175	1126
PP005	1233	1241	1225
PP007	1087	1126	1074
PP008	1192	1205	1169
PP009	1177	1168	1168
PP010	1232	1235	1203
PP013	1122	1111	1070
PP014	1102	1115	1072
PP015	1082	1100	1069
mean	1171	1179	1150
SEM	22	20	23

Right Cathode Configuration -1.5T			
Cycle duration (ms)			
ID	Ctrl	Stim	Next
PP001	1320	1339	1315
PP003	1181	1196	1169
PP004	1163	1178	1137
PP005	1248	1263	1239
PP007	1061	1077	1046
PP008	1178	1178	1166
PP009	1166	1177	1164
PP010	1268	1270	1223
PP013	1078	1071	1055
PP014	1107	1106	1085
PP015	1102	1105	1082
mean	1170	1178	1153
SEM	25	26	25

Left Cathode Configuration -1T			
**Cycle duration (ms)**			
**ID**	Ctrl	Stim	Next
**PP001**	1277	1259	1275
**PP003**	1200	1215	1174
**PP004**	1156	1146	1127
**PP005**	1248	1248	1255
**PP007**	1034	1033	1048
**PP012**	1184	1132	1147
**PP013**	1096	1065	1090
**PP014**	1114	1101	1123
**PP015**	1135	1113	1143
**mean**	1160	1146	1153
**SEM**	25	26	24

Left Cathode Configuration -1.5T			
**Cycle duration (ms)**			
**ID**	Ctrl	Stim	Next
**PP001**	1262	1228	1249
**PP003**	1204	1218	1183
**PP005**	1251	1254	1267
**PP007**	1059	1052	1060
**PP013**	1099	1063	1093
**PP014**	1111	1077	1106
**PP015**	1123	1123	1136
**mean**	1159	1145	1156
**SEM**	30	33	30
